# Effects of nutritional and ambient oxygen condition on biofilm formation in *Mycobacterium avium* subsp. *hominissuis* via altered glycolipid expression

**DOI:** 10.1038/srep41775

**Published:** 2017-02-03

**Authors:** Takahiro Totani, Yukiko Nishiuchi, Yoshitaka Tateishi, Yutaka Yoshida, Hiromi Kitanaka, Mamiko Niki, Yukihiro Kaneko, Sohkichi Matsumoto

**Affiliations:** 1 Department of Bacteriology, Osaka City University Graduate School of Medicine, 1-4-3 Asahi-machi, Abeno-ku, Osaka 545-8585, Japan; 2 Toneyama Institute for Tuberculosis Research, Osaka City University Medical School, 5-1-1, Toneyama, Toyonaka, Osaka, 560-8552, Japan; 3 Department of Bacteriology, Graduate School of Medical and Dental Sciences, Niigata University, 1-757, Asahimachi-Dori, Chuo-ku, Niigata, 951-8510, Japan; 4 Department of Structural Pathology, Graduate School of Medical and Dental Sciences, Niigata University, 1-757 Asahimachi-Dori, Chuo-ku, Niigata, 951-8510, Japan

## Abstract

*Mycobacterium avium* subsp. *hominissuis* (MAH) is the major causative agent of nontuberculous mycobacteriosis, the representative case of environment-related infectious diseases the incidence of which is increasing in industrialized countries. MAH is found in biofilm in drinking water distribution system and residential environments. We investigated the effect of gaseous and nutritional conditions, and the role of glycopeptidolipids (GPLs) on biofilm-like pellicle formation in MAH. Pellicle formation was observed under 5% oxygen in Middlebrook 7H9 broth containing 0.2% glycerol and 10% albumin-dextrose-catalase enrichment but not under normoxia or in nutrient-poor media. An analysis of 17 environmental isolates revealed that hypoxia (5% oxygen) preferentially enhanced pellicle formation both in plastic plates and in glass tubes, compared with hypercapnia (5% carbon dioxide). Wild-type strains (WT) developed much thicker pellicles than GPL-deficient rough mutants (RM). WT bacterial cells distributed randomly and individually in contrast to that RM cells positioned linearly in a definite order. Exogenous supplementation of GPLs thickened the pellicles of RM, resulting in a similar morphological pattern to WT. These data suggest a significant implication of eutrophication and hypoxia in biofilm-like pellicle formation, and a functional role of GPLs on development of pellicles in MAH.

Recently, nontuberculous pathogenic mycobacteria have received increasing attention as emerging etiological agents of infectious diseases, because *Mycobacterium avium* complex (MAC) disease patients are rapidly increasing in industrialized countries such as the United States and Japan^1^,^2^. The increased MAC disease population contains immunocompetent patients without clear cellular immunodeficiency, rather than HIV-infected patients, to whom much attention was paid a few decades ago^3^. In 2014, a national survey in Japan showed that the disease incidence of nontuberculous mycobacteria has increased to 15 cases per 100,000 population, which is 3-fold higher than that in 2004^2^. Recently, we showed that bathtub inlets and showerheads in the residential bathrooms of MAC lung disease patients are environmental sources of MAC by proving that the genotype of *Mycobacterium avium* subsp. *hominissuis* (MAH) in these sources was identical to that in patients’ sputum isolates^4^,^5^,^6^. This finding has been supported by the following study by other groups^7^,^8^. In order to find a better strategy to control MAH infection, it is necessary to understand the ecology of MAH in detail.

Generally, bacteria form biofilm by sensing various nutritional, gaseous, osmolar, and microfluidic conditions in the environment^9^. In fact, *M. avium* and other nontuberculous mycobacteria form biofilm in drinking water pipes^10^,^11^. Furthermore, various kinds of mycobacteria including MAH and other nontuberculous mycobacteria, as well as *M. tuberculosis* are known to form pellicles in experimental culture conditions, a sort of the biofilm structure consisting of clustered bacterial cells with self-produced matrix^12^,^13^,^14^,^15^,^16^,^17^. However, there is little knowledge of the optimal condition of biofilm formation in mycobacteria. One report has shown that biofilm formation by rapidly growing mycobacteria, but not slowly growing mycobacteria including MAH, on polycarbonate or stainless steel plates is enhanced by eutrophy^12^. However, the number of *M. avium* in raw waters is correlated with turbidity, suggesting *M. avium* cells bind to colloidal or suspended particles^10^. Furthermore, the effects of gaseous conditions on biofilm formation by MAH have not been fully elucidated, although adapting to hypoxia is characteristic to mycobacteria such as dormancy^18^, caseous granuloma^19^ and DosSR O_2_-sensing two component system^20^,^21^,^22^. It is of great interest to elucidate the optimal environmental conditions for biofilm formation in MAH.

Microbial surface molecules are important for attaching to matrix surface and forming microcolonies. In nontuberculous mycobacteria including *M. avium* and *M. smegmatis*, glycopeptidolipids (GPLs) are located in the outermost surface layer of the envelope^23^,^24^,^25^,^26^. The biological significance of GPLs was demonstrated by showing that GPL-deficient mutants exhibit changes in colony morphology (rough mutants) and impaired sliding motility on the water surface layer^23^,^24^. In *M. avium*, mutants of genes encoding GPL synthesis enzymes, as well as those encoding tricarboxylic acid cycle and other hypothetical membrane proteins impair pellicle formation, invasiveness in human bronchial epithelial cells, and virulence in mice when infected by aerosol^27^,^28^. These findings suggest that GPLs may have a critical role in MAH during biofilm growth, compared with planktonic growth. Proteomic analyses also revealed that bacteria in biofilm have altered metabolic activities compared with planktonic growth^16^,^29^. It is tempting to assume that MAH cells in biofilm and planktonic MAH cells have different lipid profiles.

In order to understand the role of environmental conditions in biofilm formation by MAH, we investigated several nutritional and ambient gaseous conditions by profiling the formation of biofilm-like pellicles in environmental MAH isolates from patients’ bathrooms, the causative place of infection of MAH to humans^4^,^6^. We also determined the role of cell wall components, e.g. mycobacterial cell wall lipids, including GPLs and other glycolipids, on pellicle formation by MAH.

## Results

### Pellicle formation is enhanced in eutrophic condition under hypoxia in MAH

**Figure 1:**
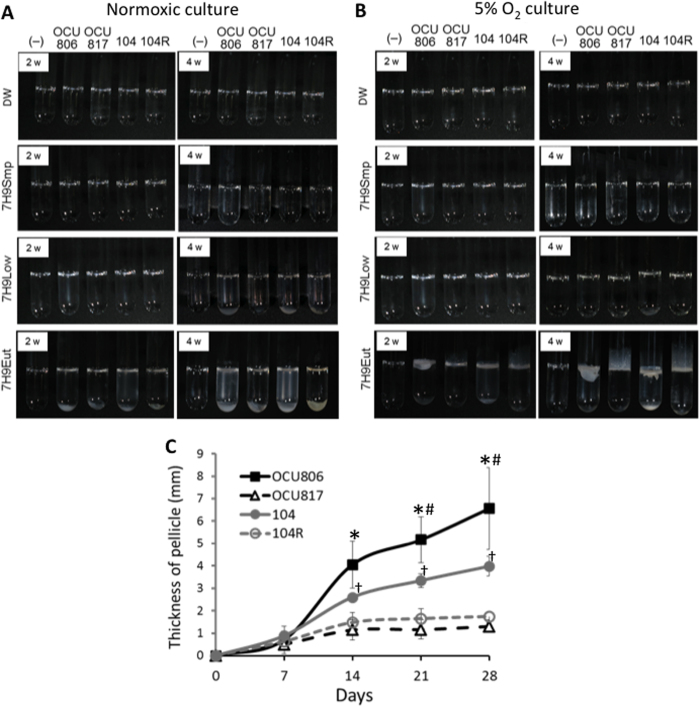
Observed difference in thickness and amount of pellicles between wild-type and rough mutant MAH strains. (**A**,**B**) Pellicle formation in MAH. Bacteria were cultured in glass tubes for 4 weeks in normoxia (**A**) or 5% O_2_ condition (**B**) in distilled water (DW), simple 7H9 broth without supplementation of carbon and nitrogen sources (7H9Smp), 7H9Smp supplemented with 0.02% glycerol and 1% ADC enrichment (7H9Low), or 7H9Smp supplemented with 0.2% glycerol and 10% ADC enrichment (7H9Eut). (−) = no bacteria, 806 = MAH OCU806, 817 = MAH OCU817, 104 = MAH 104, and 104 R = MAH 104 R. (**C**) Time-course change of thickness of the pellicles in MAH cultured in 7H9Eut under 5% O_2_ condition. Data were expressed as means ± S.D from three independent experiments. *P < 0.01 versus the OCU817 group. ^#^*P* < 0.05 versus the 104 group. ^†^*P* < 0.05 versus the 104 R group.

In order to investigate the environmental factors that regulate pellicle formation by MAH, we analysed the effects of nutritional and ambient gaseous conditions in four MAH strains, including an environmental isolate, MAH OCU806, the standard reference strain MAH 104, and their rough typed natural mutants named MAH OCU817 and MAH 104 R, respectively (Supplementary Fig. S1). Generally, rough typed mutants are known to be deficient in GPL production^26^. We confirmed that both MAH OCU817 and MAH 104 R were GPL-deficient strains and that the genotypes of these rough mutants were identical to each parent strain, as demonstrated by pulse-field gel electrophoresis. Biofilm formation by *M. avium* in drinking water pipes^10^,^11^ led us to predict that oligotrophy might be the optimal nutritional condition for biofilm formation in MAH. Thus, we analyzed four kinds of nutrient conditions; distilled water (DW), simple 7H9 medium without supplementation of carbon and nitrogen sources (7H9Smp), 7H9 medium supplemented with 0.02% glycerol and 1% albumin-dextrose-catalase (ADC) enrichment (7H9Low), and 7H9 medium supplemented with 0.2% glycerol and 10% ADC (7H9Eut). In addition, we analysed normoxic (21% O_2_) and hypoxic (5% O_2_) conditions for biofilm-like pellicle formation by MAH. Unpredictably, we found that MAH formed pellicles only under eutrophic (7H9Eut) and hypoxic conditions. Neither did MAH form pellicles under eutrophic and normoxic condition nor under oligotrophic conditions including DW (Fig. 1A,B). These data show that both eutrophy and hypoxia are necessary factors for pellicle formation in MAH.

Interestingly, we also found remarkable differences in thickness of pellicles between the wild-type strains (MAH OCU806 and MAH 104) and the GPL-deficient rough mutants (MAH OCU817 and MAH 104 R) (Fig. 1B,C) when grown under eutrophic (7H9Eut) and hypoxic condition. The former formed thick pellicles on the air-liquid interface. By contrast, the latter formed thin pellicles, and some part of the pellicles grew slightly upward along the walls of the glass tubes (Fig. 1B). These data suggest that GPLs play a significant role in the development of pellicles by MAH at the air-liquid interface.

Additionally, pellicle formation by MAH 104 cells was lower than that by MAH OCU806 cells (Fig. 1C). This is relevant to the incapability to increase GPL production in pellicle bacteria compared with the high GPL-producing strain, MAH OCU806 (described later).

### Hypoxia rather than hypercapnia enhances pellicle formation by environmental MAH isolates

Several reports of the induction of dormancy, granuloma formation and pellicle formation suggest that ambient gaseous conditions, such as hypoxia and hypercapnia, may be involved in changing the growth mode of mycobacteria^14^,^18^,^19^,^20^,^21^,^22^. To elucidate the effect of gaseous conditions on pellicle formation, we compared the profiles of pellicle formation by 17 environmental MAH strains, which were isolated from bathrooms used by MAC lung disease patients, between hypoxia (5% O_2_) and hypercapnia (5% CO_2_) (Table 1, Supplementary Table S1). In hypoxia, 10 isolates formed obvious pellicles by d 14, and additional 3 isolates formed pellicles by d 32 in polystyrene plates. In hypercapnia, no isolates formed pellicles by d 14, and only 5 isolates formed pellicles by d 32 in polystyrene plates. We also cultured the isolates for 14 d in glass tubes and found that 9 isolates formed thicker pellicles under hypoxia compared with those formed under hypercapnia. Additionally, seven isolates formed pellicles in hypercapnia as thick as in hypoxia, while one isolate formed thicker pellicles in hypercapnia than in hypoxia. (Supplementary Fig. S2). We found that hypercapnia enhanced pellicle formation in *M. bovis* Bacillus Calmette-Guérin (BCG), which is in accordance with a study of *M. tuberculosis* by Ojha (Supplementary Fig. S3)^14^. On the other hand, hypoxia did not enhance pellicle formation by *M. bovis* BCG. These data suggest that hypoxia preferentially enhances pellicle formation by MAH, and that the different effects of hypercapnia on pellicle formation between MAH and *M. tuberculosis* complex rely on the difference of mycobacterial species.

### Ultrastructural difference of pellicle between wild-type strains and rough mutants

**Figure 2:**
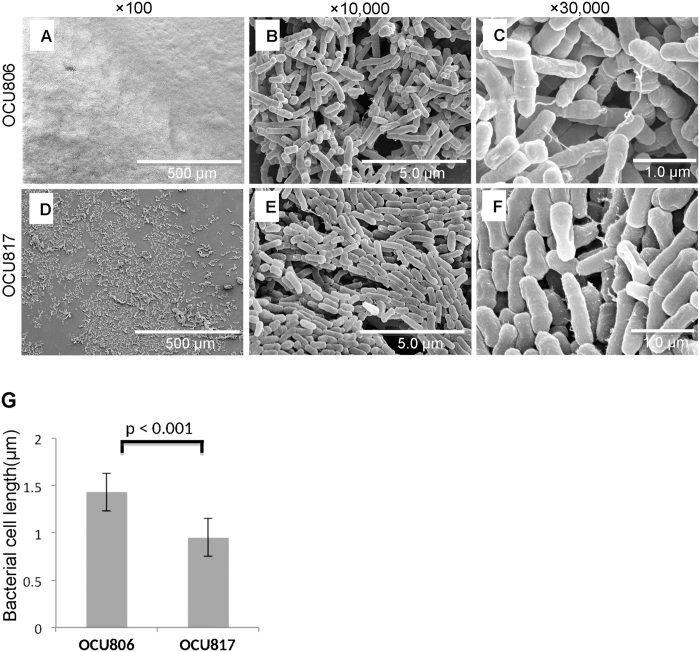
Ultrastructure of pellicles formed by wild-type and rough mutant MAH cells. (**A–F**) Pellicles formed on air-liquid interface of 7H9Eut medium under 5% O_2_ condition at 3 weeks. High performance field is shown by enlarging area that contains bacterial clustering and extracellular matrix. (**G**) Comparison of the bacterial cell size (length of the major axis) between MAH OCU806 and MAH OCU817. Data were expressed as means ± S.D. (n = 50).

We investigated the ultrastructural difference of pellicles between wild-type strains and rough mutants using scanning electron microscopy. The GPL-producing wild-type strains MAH OCU806 and MAH 104 formed large diffuse microcolonies similar to the high biofilm-forming *M. abscessus* strain as reported previously^12^ (Fig. 2A, Supplementary Fig. S4). The bacteria clustered tightly covering all over the surface, resulting in forming robust membranous structure. These bacterial cells were distributed in skew position each other, indicating random and individual arrangement similar to that in smooth-colony strains of rapidly growing mycobacteria as reported previously^23^ (Fig. 2B,C, Supplementary Figs S4 and S5). By contrast, the non-GPL producing rough mutants MAH OCU 817 and MAH 104 R formed sparse microcolonies similar to the low biofilm-forming *M. smegmatis* strain as reported previously^12^ (Fig. 2D, Supplementary Fig. S4). These bacterial cells tended to be positioned in parallel or in the same plane each other, showing linear arrangement in a definite order (Fig. 2E,F, Supplementary Figs S4 and S5). This pattern is also similar to the arrangement of the bacterial cells of rough-colony strains of rapidly growing mycobacteria as reported previously^23^. Furthermore, the size of the cells in wild-type strains was significantly smaller than that of the cells in rough mutants (mean ± SD: 1.43 ± 0.52 μm in MAH OCU806 vs. 0.95 ± 0.19 μm in MAH OCU817, *P* < 0.001; 1.10 ± 0.26 μm in MAH 104 vs. 0.95 ± 0.22 μm in MAH 104 R, *P* < 0.001) (Fig. 2G, Supplementary Fig. S4). These data suggest the role of GPLs on the distribution and integrity of the cells in the pellicles formed by MAH.

### Role of pellicles in tolerance to disinfectants

**Figure 3:**
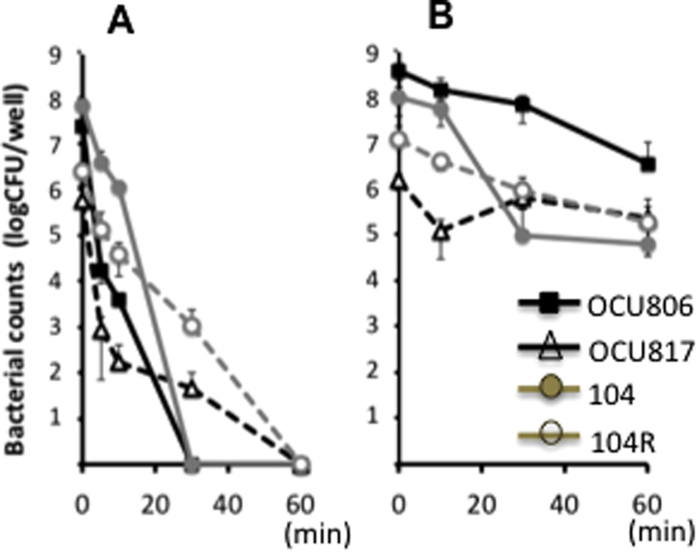
Tolerance of pellicle bacteria to sodium hypochlorite in wild-type and rough mutant MAH strains. (**A**) Planktonic bacteria. (**B**) Pellicle bacteria. First, planktonic bacteria (OD_660_ 0.5) and pellicle bacteria were prepared in 96-well plates in 7H9Eut under normoxic and 5% O_2_ condition, respectively. After removing the medium, the cells were resuspended in distilled water and treated with 1 mg/mL sodium hypochlorite. Data were expressed as means ± S.D. from four independent experiments.

Tolerance to antibiotics, disinfectants and biocides is a hallmark of biofilm bacteria^15^,^30^. In order to determine whether the MAH pellicles are really tolerant to such agents, we compared the sensitivity of planktonic bacteria and pellicle bacteria to sodium hypochlorite. Both wild-type and rough mutant planktonic cells were killed completely by 1 mg/mL sodium hypochlorite (Fig. 3A). By contrast, both wild-type and rough mutant bacterial cells in pellicles remained survived even after 60 min-exposure of 1 mg/mL sodium hypochlorite (Fig. 3B). These data suggest that MAH cells in pellicles, even non-GPL producing rough mutant cells in pellicles, are tolerant to disinfectants.

### Profile of mycobacterial lipid contents in bacteria growing in pellicles

**Figure 4:**
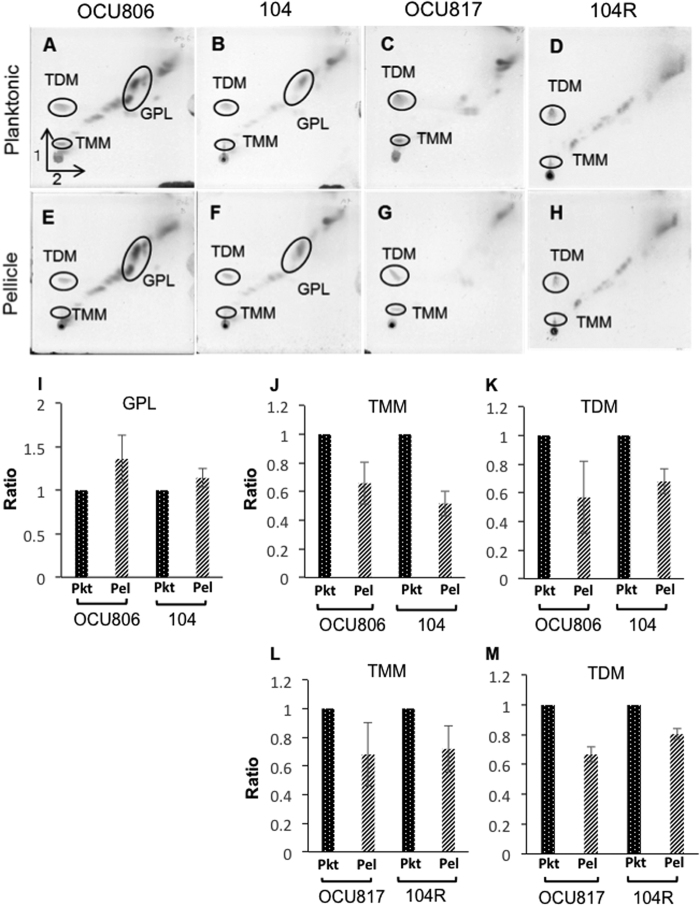
Profile of MAH glycolipids during planktonic and pellicle phases. Mycobacterial lipids were extracted from 3-week culture of planktonic bacteria (**A**‒**D**) and pellicle bacteria. (**E**‒**H**) of MAH OCU806 (**A,E**), MAH 104 (**B,F**), MAH OCU817 (**C,G**) and MAH 104 R (**D,H**), and each sample was applied on 2D-TLC. GPLs (**I**), TDM (**J,L**) and TMM (**K,M**) were quantified using image analyser. Data of pellicle bacteria were represented as the ratio of the density to the respective sample of planktonic bacteria. Data were expressed as means ± S.D from three independent experiments. Pkt = planktonic bacteria, Pel = Pellicle bacteria. See also Supplementary Fig. S6 for the mass spectrometry data to confirm the assignments of glycolipids.

In general, for most bacteria other than mycobacteria, polysaccharides are the major components of biofilms^31^. By contrast, our finding of the effect of GPLs on thickening of pellicles suggests that GPLs may be a major constituent of biofilm bacteria in pellicles as a cell wall lipid. Also, some reports have suggested that mycobacteria metabolise trehalose dimycolate (TDM), one of the major glycolipids during pellicle formation, which results in production of free mycolic acids^13^,^32^. Thus, we compared the profile of mycobacterial glycolipids between pellicle bacteria and planktonic bacteria by two-dimensional thin-layer chromatography (2D-TLC). The location of each glycolipid on TLC was confirmed by electrospray ionization mass spectrometry (ESI/MS) and matrix-assisted laser desorption/ionization time-of-flight mass spectrometry (MALDI-TOF MS) (Supplementary Figs S6 and S7). MAH OCU806 produced more GPLs (1.36 ± 0.27 fold) during pellicle growth than during planktonic growth (Fig. 4A,E,I), but the increase of GPL production in MAH 104 during pellicle growth was smaller (1.14 ± 0.21 fold) than in the case of MAH OCU806 (Fig. 4B,F,I), which corresponded to less capability of pellicle formation in MAH 104 than in MAH OCU806 as shown in Fig. 1B. Both wild-type strains and rough mutants produced smaller amounts of trehalose monomycolate (TMM) (0.66 ± 0.15 fold in MAH OCU806, 0.52 ± 0.09 fold in MAH 104, 0.68 ± 0.22 fold in MAH OCU817 and 0.72 ± 0.16 fold in MAH 104 R) and TDM (0.57 ± 0.25 fold in MAH OCU806, 0.68 ± 0.09 fold in MAH 104, 0.67 ± 0.05 fold in MAH OCU817 and 0.80 ± 0.04 fold in MAH 104 R) during pellicle growth than during planktonic growth (Fig. 4A–H,J–M). While a decrease of TDM production was first reported in *M. tuberculosis*^32^, the decrease of TMM production by pellicle bacteria was observed in mycobacteria for the first time in this study. However, we could not detect significant increase of free mycolic acids in pellicle bacteria (Supplementary Fig. S8) in contrast to a previous report for *M. tuberculosis*^32^.

### GPL determines the pattern of the pellicle phenotype

**Figure 5:**
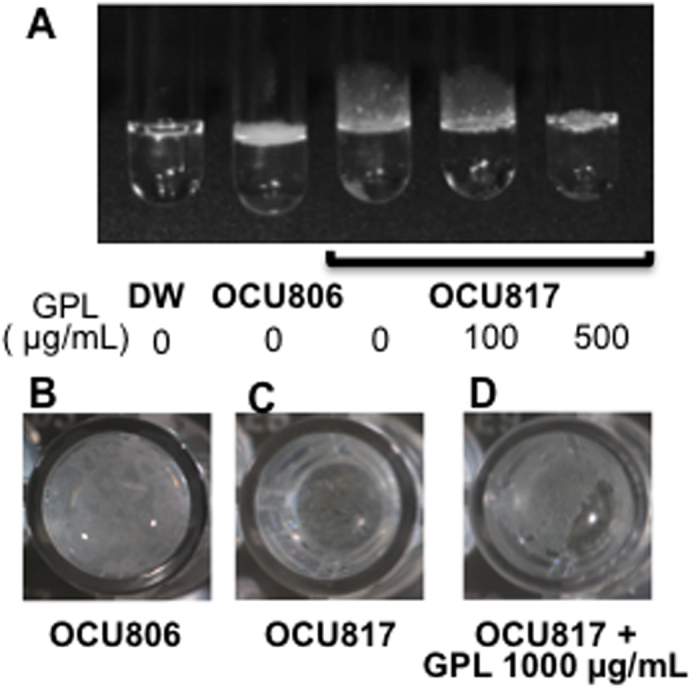
Involvement of GPLs in thickening of MAH pellicles. (**A**) Side view. A rough mutant MAH OCU817 was cultured in supplementation with GPLs (the right 2 tubes). Bacteria were cultured in 7H9Eut under 5% O_2_ condition for 5 weeks. DW = distilled water without bacteria. (**B**‒**D**) Top down view in 24-well plates. MAH OCU806 (**B**), MAH OCU817 (**C**), and MAH OCU817 culture in supplementation with GPLs (**D**). The culture method is the same as mentioned above.

In order to determine whether GPLs play a critical role in pellicle formation, we performed the exogenous supplementation of 100–1,000 μg/ml of purified GPLs to the actively growing rough mutant MAH OCU817 cells. We have purified GPLs from acetone-soluble fraction extracted from MAH OCU806, and the purity was confirmed by TLC and ESI/MS (Supplementary Fig. S9). The results showed that exogenously supplemented GPLs thickened the pellicles in MAH OCU817 so that it was similar to the pattern of the pellicles of MAH OCU806 cells (Fig. 5). These data directly show the role of GPLs in thickening of the pellicles. Furthermore, these data implicate the physical role of the amphiphilic property of GPLs on the lubricating effect that aids bacterial assembly, which has also been suggested by previous studies of mycobacteria^24^,^33^,^34^.

## Discussion

In this study, we demonstrated special characteristics of biofilm-like pellicle formation by MAH, which has emerged as an important cause of infectious diseases in industrialised countries in the last decade^1^,^2^, as follows: (1) Eutrophication and hypoxia are necessary factors for pellicle formation. (2) The presence of GPLs plays a major role in development of pellicles. (3) Factors other than GPLs are also implicated for pellicle formation by MAH, because even rough mutants were able to form thin but disinfectant-resistant pellicles. These conditions and contributing molecules for biofilm-like pellicle formation seem to be quite distinct in MAH compared with those in other bacterial species such as *Vibrio*, *Staphylococci* and *Pseudomonas*^35^,^36^,^37^, because oligotrophy and exopolysaccharides play important role in biofilm formation in such general bacteria.

Our finding of pellicle formation induced by eutrophy under hypoxia was unpredictable because *M. avium* has been shown to inhabit in drinking water^10^. However, the impact of oligotrophy on biofilm formation in *M. avium* is far from certain as shown by the fact that the bacterial number of *M. avium* grown in biofilm in water systems is positively correlated with the nutritional richness as indicated by the degree of turbidity and colloids^10^. Furthermore, several kinds of rapidly growing nontuberculous mycobacteria form biofilm on stainless steel or polycarbonate plates in eutrophy^12^. In contrast to a recent report showing pellicle formation in isotonic oligotrophic solution by using extremely high dose of MAH cells (3 × 10^8^ cells/mL)^38^, we adopt the most physiologically probable conditions by starting cultures with a low concentration of bacteria (approximately 0.03 × 10^8^ cells/mL) under hypoxia. In this condition, we confirmed that oligotrophy including distilled water and low carbon and nitrogen sources (7H9Smp, 7H9Low) did not induce pellicle formation in MAH, suggesting that eutrophy plays an important role for biofilm-like pellicle formation by MAH. Further studies are necessary to elucidate the different characteristics of biofilm formation between oligotrophy and eutrophy among various kinds of strains and species of mycobacteria.

Even in coastal geographic area, there are many hypoxic microenvironment where MAH inhabits. In fact, *M. avium* are isolated in water with hypoxic condition (dissolved oxygen content less than 10 mg/L) in natural environment^39^. Moreover, oxygen only penetrated approximately 50 μm into the biofilm in *P. aeruginosa*^40^. Thus, biofilm itself seems to produce hypoxic microenvironments. In such limited oxygen condition, bacterial cells exert transcriptomic response to keep themselves alive in biofilm^41^. The thickness of pellicles in our study is millimeter order, so the idea that most part of the inner pellicle should be microenvironmentally hypoxic is reasonable. Furthermore, pellicle has been long recognized as a kind of phenotypical characteristic in mycobacteria, which reflects the migration for the advantageous niche for aerobes due to increased accessibility to oxygen as shown in *M. tuberculosis* and *M. smegmatis* grown in experimental settings^15^. Taking all things into consideration, our finding of pellicle formation under hypoxia in MAH suggests the importance of hypoxia on adaption for ecological niches including biofilm formation in MAH.

Mycobacteria are well-known to form granuloma whereby mycobacteria reside as a persistent cell under hypoxic condition for decades until reactivation^19^. DosSR, one of the representative O_2_ sensing system in mycobacteria, induces the expression of dozens of genes under hypoxic condition to enable the bacteria to adapt to environmental stress^20^,^22^. *M. tuberculosis* has two kinds of sensors DosS and DosT, which work as a redox sensor and a hypoxia sensor, respectively. However, *M. avium* has only a DosT homologue, and this homologue is phylogenetically distinct from both DosT and DosS of *M. tuberculosis*^21^. Such difference of DosSR system may explain why pellicle formation was preferentially induced by hypoxia in MAH as shown in this study, which is in contrast to the data of Ojha, who observed enhanced pellicle formation under hypercapnia in *M. tuberculosis*^14^.

Our data of pellicle formation in GPL-deficient rough mutants, especially slightly upward growth along the wall of glass tubes, suggest the different aspects of substratum attachment, intercellular aggregation and sliding motility between MAH and *M. smegmatis*. In *M. smegmatis*, loss of GPLs is directly correlated with these biofilm-related cellular behaviors^25^,^42^,^43^. By contrast, such direct relationship does not seem to be applicable in nontuberculous mycobacteria other than *M. smegmatis*. For example, rough mutants of MAH have been reported to increase the attachment to the glass surface by forming flocculant patterns of binding^44^. Similarly, such increased attachment to the glass surface with decreased sliding motility has also been found in other nontuberculous mycobacteria^23^. The preferential attachment to the glass surface may result in the upward growth of the pellicles on the glass tubes in rough mutants by the change of amphiphilic moiety through loss of GPLs^25^,^33^. On the other hand, the pellicle was formed by cell-cell clustering by direct interaction between bacterial cells as shown in Fig. 2, resulting in the growth of macrocolonies on the air-liquid interface as floating aggregates. Taking all things into consideration, we can speculate that the influence of loss of GPLs on each kind of biofilm-related behaviors varies between mycobacterial species. Further studies are necessary to elucidate the detailed relationship between GPLs and substratum attachment in MAH.

Our finding that rough mutants formed thin, but biocide-resistant, pellicles not only suggests a major role of GPLs in development of biofilm-like pellicles but also possible contributing factors to pellicle formation other than GPLs by MAH. GPLs are located at the outermost surface of the cell envelope, and GPLs are amphiphilic molecules that are composed of hydrophilic polysaccharide capsule and lipophilic fatty acid moiety^25^,^33^. Any alteration of lipophilic characteristics of outermost cell surface layer resulting from the loss of GPLs may affect cell-cell and cell-matrix lubrication, as well as integrity of cell population^25^,^33^. The loss of GPLs in rough mutants alters the amphiphilic characteristics of the surface of the cell envelope, possibly resulting in increased friction at the air-liquid interface. This notion is supported by previous reports showing that the loss of GPLs in nontuberculous mycobacteria alters sliding motility at the air-liquid interface^23^,^24^,^25^, as well as by the difference in cell aggregation between wild-type strains and rough mutants in this study. Furthermore, the smaller difference of the increase of GPLs in the pellicles of MAH 104 seems to be relevant to its diminished ability to form pellicles (as observed by more fragile and thinner pellicles that had a softer membranous ultrastructure), compared with the high pellicle-producing strain MAH OCU806. Taken together, we suggest that amphiphilicity is one of the important physiological characteristics for biofilm development in MAH.

In MAH, causal relationship has not been proven yet between the ability of GPL production and virulence in contrast to the case of *M. abscessus* in which rough mutants are hypervirulent by producing massive serpentine cords^45^. Although one paper suggested the hypervirulence of rough mutants in MAH for chickens^46^, most of other papers do not support such notion experimentally. For example, MAH 101 rough mutants are reported not to grow in beige mice^47^, and the virulence of MAH 104 does not differ between wild-type strains and rough mutants in C57BL/6 mice^48^. These data suggest that the relationship between the loss of GPLs (rough mutants) and virulence is far from simple in MAH compared with *M. abscessus*. Thus, it is beyond the scope of this study to elucidate the significance of different patterns of pellicle formation in pathogenesis of human MAC disease between wild-type strains and rough mutants. On the other hand, GPLs induce humoral immune response in MAC disease patients in correlation with the range of lung lesion, suggesting some involvement of GPLs in pathogenesis of human MAC disease^49^. Taken all things together, it seems to be difficult to assume the simple and direct relationship between the loss of GPLs and acquisition of virulence in MAH in contrast to the case of *M. abscessus*.

In addition to the increase of GPLs by MAH OCU806 cells in pellicles, we demonstrated a direct and critical role of GPLs in pellicle development by exogenous supplementation of GPLs on the rough mutant MAH OCU817 cells, which increased pellicle formation. This supports the notion that the outermost molecules of the cells are important for pellicle development, which is similar to the role of extrapolysaccharides in various kinds of general bacteria^31^,^35^,^36^.

On the other hand, GPLs do not seem to be the only determinant of biofilm formation by MAH because rough mutants also formed biocide-tolerant pellicles, and not all environmental isolates formed pellicles in this study (Table 1, Supplementary Table S1). In addition to that, MAH envelope contains a range of characteristic bioactive glycolipids other than GPLs such as TDM, lipoarabinomannan and phosphatidylinositol mannoside. Similar to GPLs, these glycolipids are also antigenic in hosts^50^,^51^. Therefore, these outermost bioactive molecules may also contribute to pellicle formation by MAH.

The biofilm-like pellicle formation by *M. tuberculosis*, which does not synthesize GPLs, is reported to depend on the synthesis of mycolic acids, especially ketomycolic acid^13^,^14^,^15^. We also found a decrease of TDM and TMM in pellicle bacteria compared with those in planktonic bacteria, which suggests the activation of mycobacterial lipid metabolism during pellicle growth. This finding is consistent with the increased degradation of TDM that was reported by Ojha^32^, although we were unable to detect an increase of free mycolic acids, possibly because we used a different method to examine GPL biosynthesis, i.e., we analysed the TDM level in pellicles after 3 weeks, while Ojha incubated *M. tuberculosis* cells in the presence of ^14^C-acetate for a short (24-h) period^14^.

Most previous studies of the effect of chlorine disinfectant on *M. avium* are limited on planktonic cells^52^,^53^,^54^,^55^,^56^. Notably, we found that MAH cells in pellicles were highly tolerant to 1 h-exposure of 1 mg/mL sodium hypochlorite. This suggests that there is a risk of providing a favorable environment for the growth and persistence of MAH by such disinfectants through microbial substitution^57^.

In summary, we determined the specific condition of biofilm-like pellicle formation in MAH as eutrophy and hypoxia. We also elucidated the critical role of GPLs in development of pellicles, as evidenced by the different phenotypic patterns of biofilm-like pellicles between wild-type strains and non-GPL producing rough mutants, as assessed by ultrastructural examinations and lipid profiling. These findings provide a new insight into the adaptation of MAH to human and animal hosts as well as natural environments by exploiting crucial amphiphilic surface molecules, namely GPLs. We hope our findings provide basic knowledge for establishing better strategies for the eradication of MAC disease via multi-directional approach, which includes the development of better pharmaceutics, improved hygiene, and advances in clinical medicine.

## Methods

### Bacterial strains

MAH OCU806 was isolated from a bathtub inlet in the residence of a pulmonary MAC disease patient. MAH 104 is a reference strain derived from an AIDS patient^58^. MAH OCU817 and MAH 104 R are naturally occurring rough mutants of MAH OCU806 and MAH 104, respectively. Other environmental strains used in this study were isolated from bathrooms (bathtub inlets, showerheads, bathtub water, and drain outlets) of the pulmonary MAC disease outpatients at Toneyama National Hospital (Toyonaka, Osaka, Japan) between 2004 and 2008.

### Bacterial culture and pellicle assay

Bacteria were precultured in Middlebrook 7H9 supplemented with 0.2% glycerol and 10% ADC. Bacterial cells were collected by centrifugation at 2,330× *g* for 15 min at 4 °C followed by washing twice by 1 mL of distilled water by centrifugation at 18,000× *g* for 2 min at 4 °C. The bacterial suspension was adjusted to an optical density at 660 nm (OD_660_) of 0.1 and diluted 30-fold. The diluted bacterial suspension was inoculated on 96-well plates (200 μL/well), 24-well plates (1 mL/well), or screwless aluminum-capped glass tubes (2 mL/tube), and then incubated at 37 °C under static condition. We performed the experiments of pellicle formation with the lid not tightly closed to enable the air to pass for equilibration of the gaseous condition between outside and inside the culture tubes. For auxotrophic assays for pellicle formation, four culture media were investigated; DW, 7H9Smp, 7H9Low, and 7H9Eut (see the Results for details). All of the culture experiments were performed at 37 °C under static condition. To investigate the gaseous conditions on pellicle formation, bacteria were cultured under hypoxic or hypercapnic (5% O_2_ or 5% CO_2_) (APM-30D, ASTEC, Tokyo, Japan), or normoxic conditions (BR-3000LF, TITIEC, Tokyo, Japan). Photos were taken every 7 d for morphological evaluation of the pellicles. Quantification of the formed pellicles was performed by measuring thickness directly by a ruler, because crystal violet staining is considered not suitable to compare the biomass of pellicles, as this stains only the cells that are attached to a substratum. Data were compared by the unpaired two-tailed Student’s *t*-test at each timepoint between groups.

### Preparation of pellicles for scanning electron microscopy

First, coverslips were slipped under pellicles of bacteria grown in 24-well plates. The pellicle-mounted coverslips were prefixed in 2.5% glutaraldehyde in 0.1 M phosphate buffer (PB; pH 7.4), for 10 min and rinsed thrice with PB. Then, the samples were fixed again with 2.5% glutaraldehyde for 1 h and rinsed thrice with PB. The samples were fixed again by 1% (w/v) osmium tetroxide in PB for 1 h. Subsequently, the samples were rinsed thrice with PB and dehydrated with increasing concentrations of ethanol (30%, 50%, 70%, 90%, 99%, and 100%). Then, the dehydrated samples were soaked in isoamyl acetate, and the samples were dried at critical point drier (HCP-2; Hitachi Ltd., Tokyo, Japan), followed by coating with an 8:2 platinum-palladium alloy using an ion sputter (E-1030; Hitachi Ltd., Tokyo, Japan). The resultant coat was 12 nm thick. The samples were observed by a scanning electron microscope (S4700; Hitachi Ltd., Tokyo, Japan). Bacterial size was compared by measuring the length of the major axis of 50 cells per strain. Data were compared by the unpaired two-tailed Student’s *t*-test. Values were reported as mean ± SD. The alignment of the bacterial cells were compared by the proportion of the surrounding cells in skew position against a randomly-selected cell at 12 loci in the SEM picture (×10,000), and data were compared by the χ^2^ square test with Yate’s continuity correction (Supplementary Fig. S5). Significant difference was set at *P* < 0.05.

### Disinfectant resistance assay

To obtain pellicle bacteria, a 200-μL culture was started from a 30-fold dilution of logarithmically growing cells that were adjusted to an OD_660_ of 0.1, and continued for 2 weeks at 37 °C in 7H9Eut under 5% O_2_ in 96-well flat bottom plates. After removing the culture medium, formed pellicles and cells attached to the plate walls were resuspeneded in DW and treated with 250 μL of 1 mg/mL sodium hypochlorite for 0, 5, 10, 30, and 60 min at room temperature. The reaction was stopped by neutralisation with 50 μL of 30 mM sodium thiosulfate in 0.1 M phosphate buffer (pH 7.5). The bactericidal effect was evaluated by counting colony-forming units per well. The cells in the neutralised solution and those on the plate walls were collected using a cotton swab and transferred to a new tube as a cell suspension. The cell suspensions were 10-fold serially diluted and inoculated onto Middlebrook 7H11 agar plates containing 0.5% glycerol and 10% oleic acid-ADC enrichment, and cultured at 37 °C for 1 week. The colonies were counted using a stereo-microscope. Planktonic cells were cultured in 7H9Eut medium with shaking at 100 rpm. When the OD_660_ reached 0.5, 200 μL of the culture was transferred to a 96-well flat bottom plate. After removing the culture medium, the cells were resuspended in DW and exposed to 1 mg/mL sodium hypochlorite for 0, 5, 10, 30, and 60 min at room temperature. Neutralising reactions with sodium thiosulfate, and counts of surviving cells were performed as described above.

### Analysis of lipids

Planktonic cells were grown in 7H9Eut media at 37 °C under normoxic condition for 3 weeks, and harvested by centrifugation at 2,330× *g* for 15 min at 4 °C followed by washing twice with DW. Pellicles were allowed to form in 7H9Eut medium at 37 °C under 5% O_2_ condition for 3 weeks. Bacterial cells in the pellicles were harvested and washed twice with DW. After sterilizing at 90 °C for 20 min, the samples were lyophilised and their dry weight was measured. The bacterial cells were disrupted using a sonicator (Branson Sonifier, Tokyo, Japan) and total lipids were extracted with CHCl_3_:MeOH:DW (10:5:2 v/v/v) using the Folch procedure. The amount of total lipids derived from 1 mg of dried bacterial cells was applied to a HPTLC Silica gel 60 thin-layer chromatograph (TLC; Merck, Kenilworth, NJ, USA), and two-dimensional TLC was performed using solvent system A (solvent 1, CHCl_3_:MeOH:DW, 100:14:0.8 (v/v/v); solvent 2, CHCl_3_:acetone:MeOH:DW, 50:60:2.5:3 (v/v/v/v)) for GPLs, TDM, and TMM analyses, or using solvent system B (solvent 1, CHCl_3_:MeOH, 96: 4 (v/v); solvent 2, toluene:acetone, 80:20 (v/v)) for free mycolic acids. The plates were colorised by 20% (v/v) sulfuric acid in ethanol and heated at 165 °C for 5–10 min. The chromatograph pattern was scanned by ImageQuant LAS 4000 system, and quantified according to the manufacturer’s instructions (GE Healthcare Life Sciences, Little Chalfont, UK). To confirm the assignment of TMM, the molecular weight of the scraped samples in each spot was measured by ESI/MS by using an ESI probe in the positive ion mode (AccuTOF 4 G LC-plus; JEOL, Tokyo, Jaopan) (Supplementary Fig. S6). To confirm the assignment of TDM, the molecular weight of the scraped samples were analysed by a Ultraflex III-MALDI-TOF Mass Spectrometer (Bruker Daltonics; Leipzig, Germany) using 2,5-dihydroxybenzoic acid as a matrix in the positive ion mode (Supplementary Fig. S7). Data were expressed as the proportion of amount of each lipid in pellicle bacteria to that in planktonic bacteria.

### Collection of GPLs

A 2-L planktonic culture of strain MAH OCU806 was collected by centrifugation at 12,700× *g* for 30 min, and 8.3 g (wet weight) of bacteria were dissolved in methanol, disrupted by sonication for 24 min (38 s on/38 s off, 80% duty) (Nihon Emerson, Kanagawa, Japan). The lysate was distributed into two layers in a glass funnel by adding 240 mL of chloroform and a few milliliter of water. The lipid layer was evaporated by a rotary evaporator, and 0.79 g of crude lipids were obtained. The crude lipids were dissolved in acetone at 50 °C for 30 min and centrifuged at 1,700 × *g* for 15 min. The supernatant was distilled, and 256 mg of acetone-soluble lipids containing GPLs were obtained. After confirming the location of the spot of GPLs in a preliminary TLC assay, the lipids were developed in a solvent constituting CHCl_3_:MeOH:DW in a 100:10:0.5 (v/v/v) ratio (HPTLC Silica gel 60, Merck; Uniplate, Analtech Inc., Newark, DE, USA), and the GPL spot was scraped off the TLC plate. The sample was dissolved in a solvent constituting CHCl_3_:MeOH in a 2:1 ratio (v/v) and centrifuged at 1,700× *g* for 10 min. The supernatant was collected and centrifuged twice, and the solvent was vaporised by spraying it with nitrogen gas in a dry block bath (EYELA MG2200, Tokyo Rika Kikai. Tokyo Japan). The purity of the GPLs was confirmed by TLC and ESI/MS by AccuTOF 4 G LC-plus (JEOL, Tokyo, Japan) (Supplementary Fig. S9).

### Supplementation assay of GPLs to a rough mutant culture

Extracted GPLs were suspended in isopropanol to a concentration as 10 mg/mL, and the suspension was supplemented for growing rough mutant MAH OCU817 cells to reach a final concentration as 100, 500, and 1,000 μg/mL in 1 mL 7H9Eut in glass tubes and 24-well plates. The culture was performed in 7H9Eut under 5% O_2_ condition for 5 weeks.

## Additional Information

**How to cite this article**: Totani, T. *et al*. Effects of nutritional and ambient oxygen condition on biofilm formation in *Mycobacterium avium* subsp. *hominissuis* via altered glycolipid expression. *Sci. Rep.* **7**, 41775; doi: 10.1038/srep41775 (2017).

**Publisher's note:** Springer Nature remains neutral with regard to jurisdictional claims in published maps and institutional affiliations.

## Supplementary Material

Supplementary Information

## Figures and Tables

**Table 1:** The number of pellicle-forming MAH environmental isolates under hypoxia and hypercapnia.

			Pellicle formation ability			
			Day 14		Day 32	
Total number of isolates studied	Isolation source^a^	Colony morphology	5% O_2_	5% CO_2_	5% O_2_	5% CO_2_
17	BI: 7 BW: 6 SH: 3 DO:1	Smooth: 16 Rough: 1	10 (58.8%)	0 (0%)	13 (76.4%)	5 (29.4%)

^a^BW: bathtub water, SH: showerhead, BI: bathtub inlet, DO: drain outlet.

Pellicle formation was assayed in 24-well plastic plates in 17 clinical isolates from bathrooms of MAC lung disease patients. For detailed profile, refer to Supplementary Table S1.
