# Supplementary material for: Effects of nutritional and ambient oxygen condition on biofilm formation in Mycobacterium avium subsp. hominissuis via altered glycolipid expression
Source: Sci Rep. 2017 Feb 3;7:41775. doi: 10.1038/srep41775 (PMC5290538; doi:10.1038/srep41775)
Supplement: Supplementary Information [file srep41775-s1.pdf]

Supplementary Data

Effects of nutritional and ambient oxygen condition on biofilm formation in *Mycobacterium avium* subsp. *hominissuis* via altered glycolipid expression

Takahiro Totani, Yukiko Nishiuchi, Yoshitaka Tateishi, Yutaka Yoshida, Hiromi Kitanaka, Mamiko Niki, Yukihiro Kaneko, and Sohkichi Matsumoto

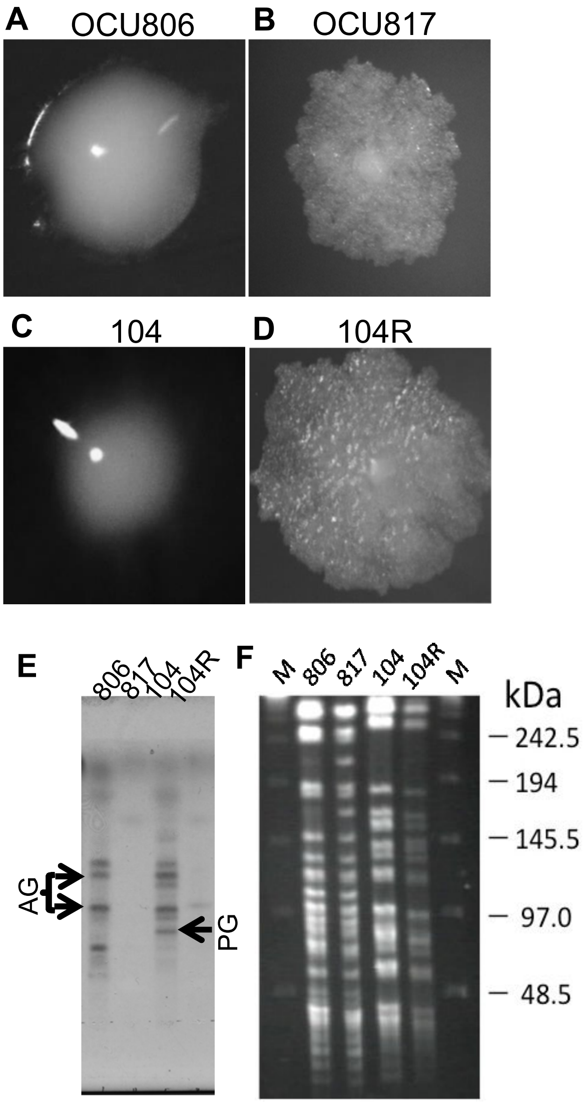

Supplementary Fig. S1. Confirmation of MAH rough mutant characteristics. (**A-D**) Colony morphologies of MAH OCU806, MAH OCU817, MAH 104, MAH 104R grown on Middlebrook 7H11 agar plates containing 0.5% glycerol, 0.06 % oleic acid and 10% ADC enrichment. (**E**) Lack of GPL production in rough mutants on TLC. AG = apolar GPLs, PG = polar GPLs. MAH OCU806 does not have PG. (**F**) Confirmation of the same genotype between rough mutants and wild-type strains by pulse-field gel electrophoresis. Genomic DNA was digested by *Xba*I.

			Pellicle formation ability			
			Day 14		Day 32	
Strain ID	Isolation source [a]	Colony morphology [b]	5% O_2	5% CO_2	5% O_2	5% CO_2
OCU401	BW	Sm	−	−	−	−
OCU433	BW	Sm	−	−	+	−
OCU446	BW	R	−	−	−	−
OCU447	SH	Sm	−	−	−	−
OCU526	BI	Sm	−	−	−	−
OCU663	DO	Sm	+	−	+	−
OCU664	BI	Sm	+	−	+	−
OCU666	BW	Sm	−	−	+	−
OCU752	BI	Sm	−	−	+	−
OCU681	BW	Sm	+	−	+	+
OCU746	SH	Sm	+	−	+	+
OCU748	BW	Sm	+	−	+	−
OCU792	BI	Sm	+	−	+	+
OCU797	SH	Sm	+	−	+	−
OCU798	BI	Sm	+	−	+	+
OCU801	BI	Sm	+	−	+	−
OCU806	BI	Sm	+	−	+	+

Supplementary Table S1. The property of environmental MAH isolates and profiles of pellicle formation ability in hypoxia and hypercapnia. Pellicle formation was assayed in 24-well plastic plates in 17 environmental isolates from bathrooms of MAC lung disease patients.

[a] BW: bathtub water, SH: showerhead, BI: bathtub inlet, DO: drain outlet.

[b] Sm: smooth, R: rough.

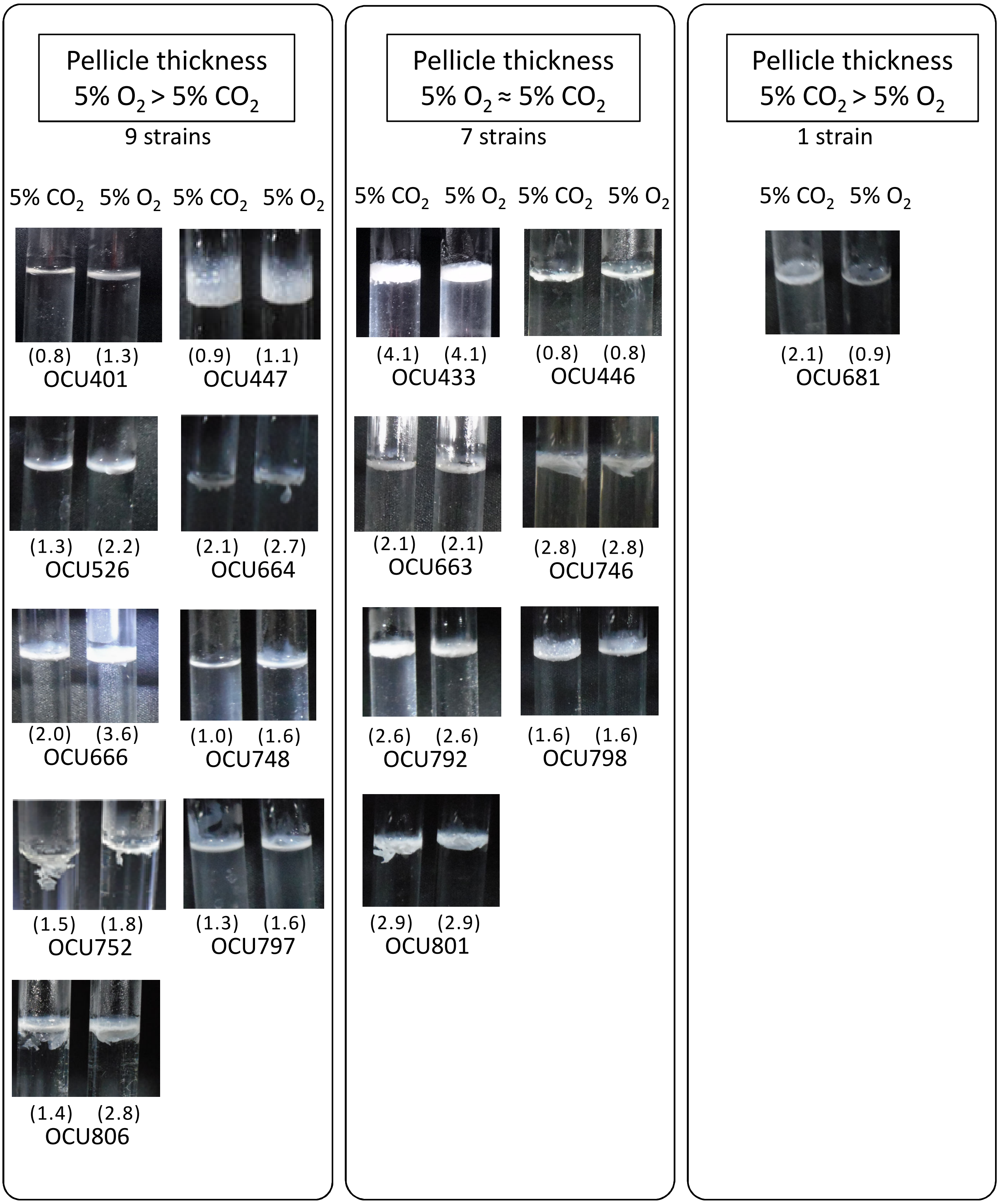

Supplementary Fig. S2. Comparison of pellicle thickness formed on air-liquid interface by 17 environmental MAH isolates between hypoxic and hypercapnic conditions in glass tubes. The MAH isolates were cultured for 2 weeks in 7H9Eut. Numbers in parenthesis indicate the thickness of pellicles (unit: mm).

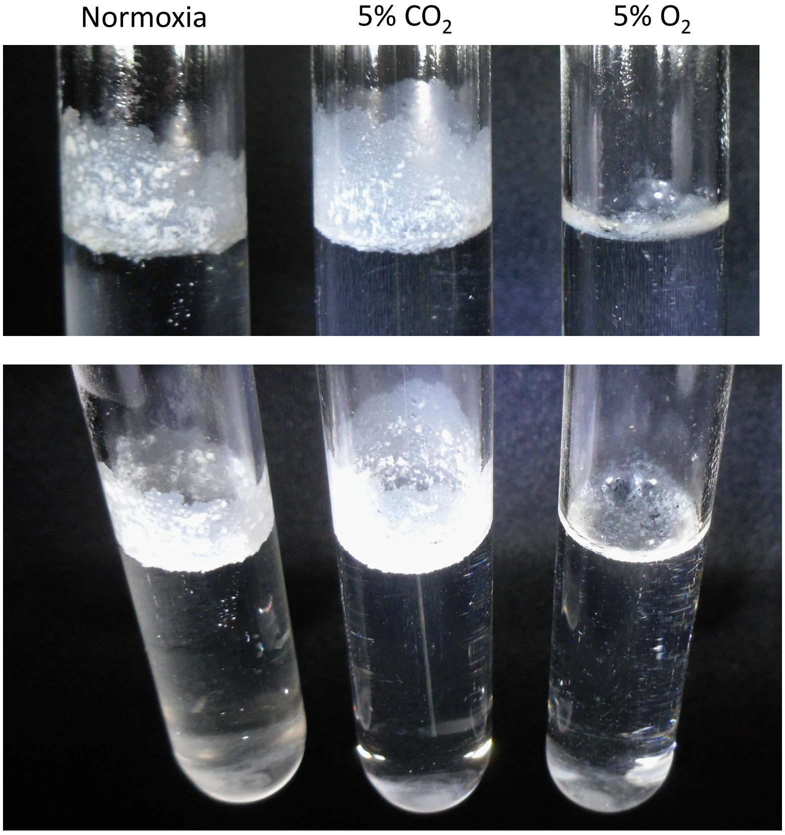

Supplementary Fig. S3. Enhancement of pellicle formation under hypercapnia but not under hypoxia in *M. bovis* bacillus Calmette-Guérin (BCG) Pasteur by 4-week culture in 7H9Eut.

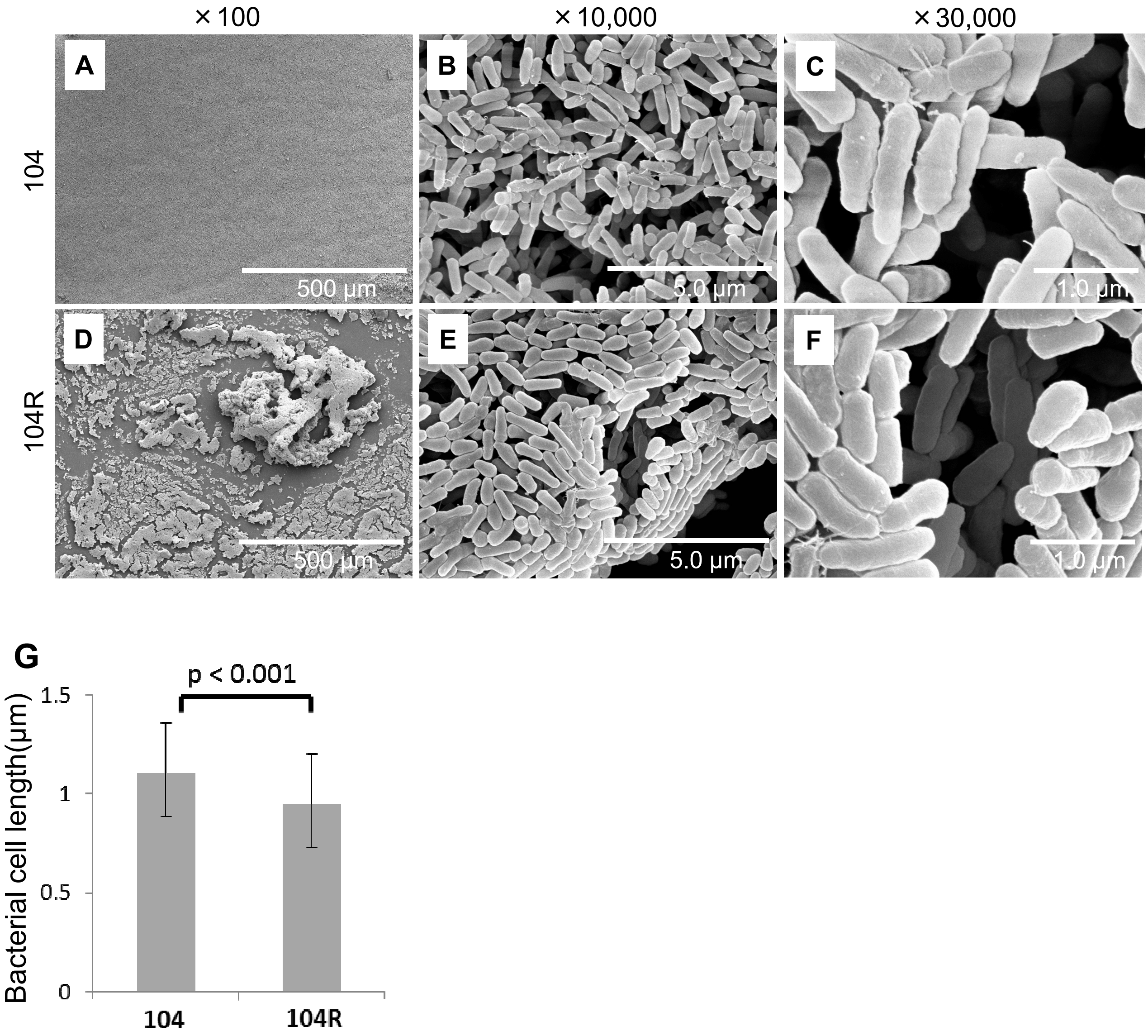

Supplementary Fig. S4. Ultrastructure of pellicles formed by MAH 104 and MAH 104R. (**A–F**) Pellicles were formed on air-liquid interface of 7H9Eut medium under 5% O_2 condition and 3-week-old pellicles were sampled for SEM observation. (**G**) Comparison of the length of the major axis of bacterial cells between MAH 104 and MAH 104R. Data were designated as means±S.D. (n = 50).

Inspector A

	OCU806	OCU817
skew (+)	76	7
skew (—)	17	89

x^2= 103.246, df= 1, *P* < 0.01

	104	104R
skew (+)	73	16
skew (—)	27	72

x^2= 54.247, df= 1, *P* < 0.01

Inspector B

	OCU806	OCU817
skew (+)	96	32
skew (—)	24	77

x^2= 80.364, df= 1, *P* < 0.01

	104	104R
skew (+)	99	27
skew (—)	21	98

x^2= 88.481, df= 1, *P* < 0.01

Inspector C

	OCU806	OCU817
skew (+)	68	19
skew (—)	27	77

x^2= 49.565, df= 1, *P* < 0.01

	104	104R
skew (+)	65	22
skew (—)	36	74

x^2= 32.615, df= 1, *P* < 0.01

Supplementary Fig. S5. Comparison of the alignment of the bacterial cells between wild-type strains and rough mutants in terms of skew position. Three inspectors who were not informed of the contents of the manuscript selected 12 bacterial cells randomly in the SEM picture (×10,000), and they individually counted the number of the surrounding cells in skew position and those in non-skew position (parallel or coplanar) evaluated by the direction of the major axis of the cells. The tables are designated as a sum of the counts at the randomly-selected 12 loci in each strain. Chi-square test was performed with Yate's continuity correction to find the difference of proportion of the cells in skew position between wild-type strains and rough mutants. df = degree of freedom.

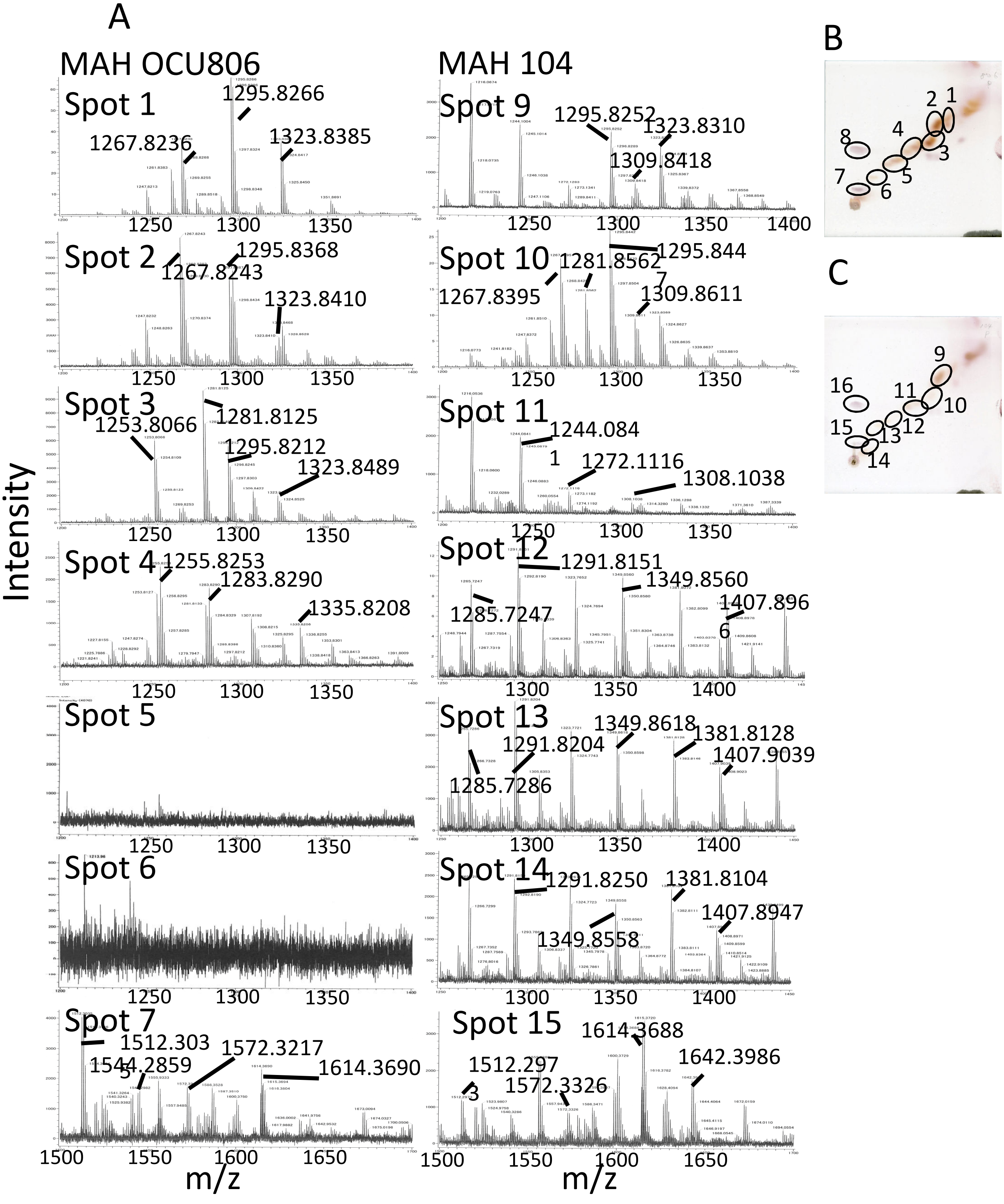

Supplementary Fig. S6.

Supplementary Fig. S6 (continued)

D

Spot No.	Glycolipid	Composition formula	Calculated	Found	Deviation (ppm)
1	Mono-*O*-acetylated GPL	$C_{68}H_{116}N_4O_{16}Na$	1267.8260	1267.8236	1.9
	Di-*O*-acetylated GPL	$C_{69}H_{116}N_4O_{17}Na$	1295.8253	1295.8266	1.0
	Di-*O*-acetylated GPL	$C_{71}H_{120}N_4O_{17}Na$	1323.8546	1323.8385	12.2
2	Mono-*O*-acetylated GPL	$C_{68}H_{116}N_4O_{16}Na$	1267.8260	1267.8243	1.3
	Di-*O*-acetylated GPL	$C_{69}H_{116}N_4O_{17}Na$	1295.8253	1295.8266	1.0
	Di-*O*-acetylated GPL	$C_{71}H_{120}N_4O_{17}Na$	1323.8546	1323.8385	12.2
3	Mono-*O*-acetylated GPL	$C_{67}H_{114}N_4O_{16}Na$	1253.8128	1253.8066	4.9
	Mono-*O*-acetylated GPL	$C_{67}H_{114}N_4O_{16}Na$	1281.8436	1281.8125	24.3
	Di-*O*-acetylated GPL	$C_{69}H_{116}N_4O_{17}Na$	1295.8253	1295.8212	3.2
	Di-*O*-acetylated GPL	$C_{71}H_{120}N_4O_{17}Na$	1323.8546	1323.8489	4.3
7	C80 α-mycoloyl TMM	$C_{92}H_{176}O_{13}Na$	1512.3009	1512.3035	1.7
	C80 wax-mycoloyl TMM	$C_{92}H_{176}O_{15}Na$	1544.2907	1544.2859	3.1
	C82 wax-mycoloyl TMM	$C_{94}H_{180}O_{15}Na$	1572.3220	1572.3217	0.2
	C85 wax-mycoloyl TMM	$C_{97}H_{186}O_{15}Na$	1614.3689	1614.3690	0.1
9	Di-*O*-acetylated GPL	$C_{69}H_{116}N_4O_{17}Na$	1295.8253	1295.8252	0.1
	Di-*O*-acetylated GPL	$C_{70}H_{118}N_4O_{17}Na$	1309.8381	1309.8418	2.8
	Di-*O*-acetylated GPL	$C_{71}H_{120}N_4O_{17}Na$	1323.8546	1323.8310	17.8
10	Mono-*O*-acetylated GPL	$C_{68}H_{116}N_4O_{16}Na$	1267.8260	1267.8395	10.7
	Mono-*O*-acetylated GPL	$C_{67}H_{114}N_4O_{16}Na$	1281.8436	1281.8562	9.8
	Di-*O*-acetylated GPL	$C_{69}H_{116}N_4O_{17}Na$	1295.8253	1295.8447	15.0
	Di-*O*-acetylated GPL	$C_{70}H_{118}N_4O_{17}Na$	1309.8381	1309.8611	17.6
15	C80 α-mycoloyl TMM	$C_{92}H_{176}O_{13}Na$	1512.3009	1512.2973	2.4
	C82 wax-mycoloyl TMM	$C_{94}H_{180}O_{15}Na$	1572.3220	1572.3326	6.7
	C85 wax-mycoloyl TMM	$C_{97}H_{186}O_{15}Na$	1614.3689	1614.3688	0.1
	C87 wax-mycoloyl TMM	$C_{99}H_{190}O_{15}Na$	1642.4002	1642.3986	1.0

Supplementary Fig. S6. Electron spray ionization mass spectrometry (ESI/MS) of glycolipids on TLC. (**A**) ESI/MS spectra of extracted glycolipids. The spots were scraped off and the glycolipids were eluted in chloroform/methanol (2:1, v/v) followed by evaporation using nitrogen gas. The samples were analyzed by ESI/MS by using an ESI probe in the positive ion mode (AccuTOF 4G LC-plus; JEOL, Tokyo, Japan). (**B,C**) TLC of total lipids extracted from planktonic MAH OCU806 (**B**) and MAH 104 cells (**C**). Spots 8 and 16 correspond to TDM as shown in **Supplementary Fig. S7**. (**D**) Composition formula, calculated mass, found mass, and deviation from calculated mass (ppm) of GPLs and TMMs identified in each spot.

References

1. Marrakchi H, Lanéelle MA, Daffé M. Mycolic acids: structures, biosynthesis, and beyond. *Chem Biol.* **21**, 67-85 (2014).
2. Fujita Y, Okamoto Y, Uenishi Y, Sunagawa M, Uchiyama T, Yano I. Molecular and supra-molecular structure related differences in toxicity and granulomatogenic activity of mycobacterial cord factor in mice. *Microb Pathog.* **43**, 10-21 (2007).

A

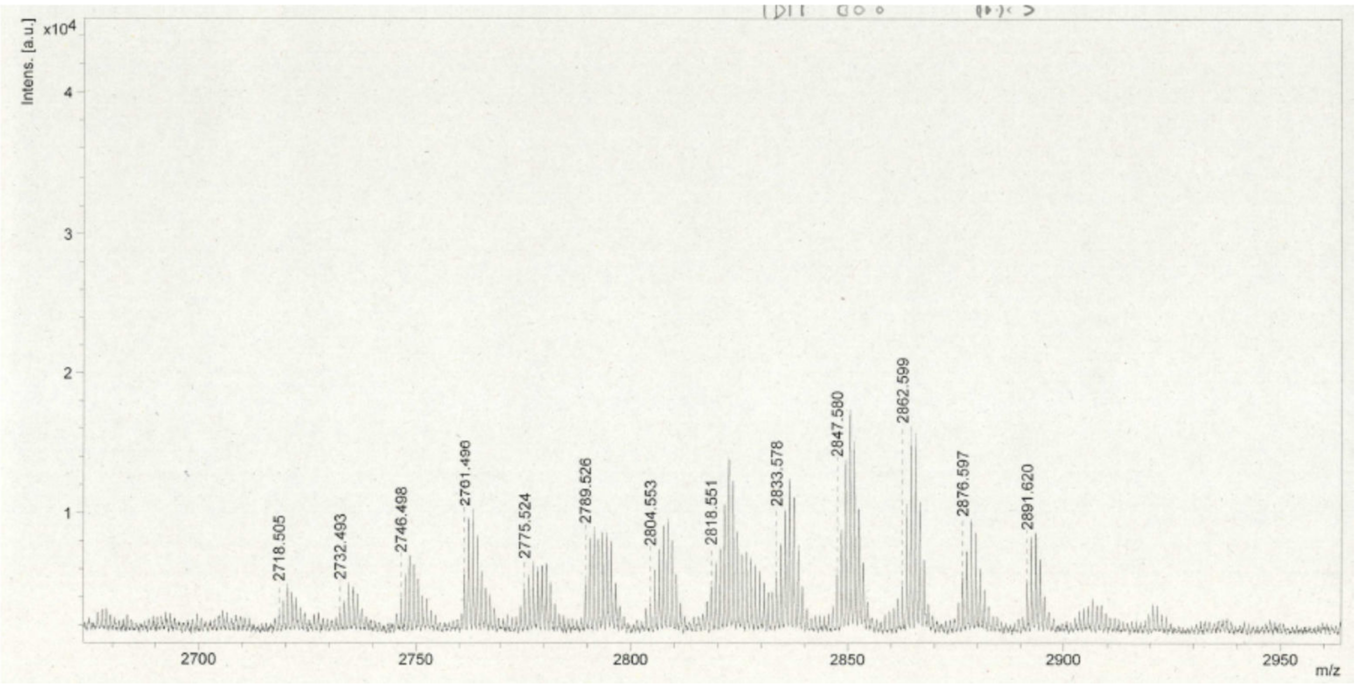

B

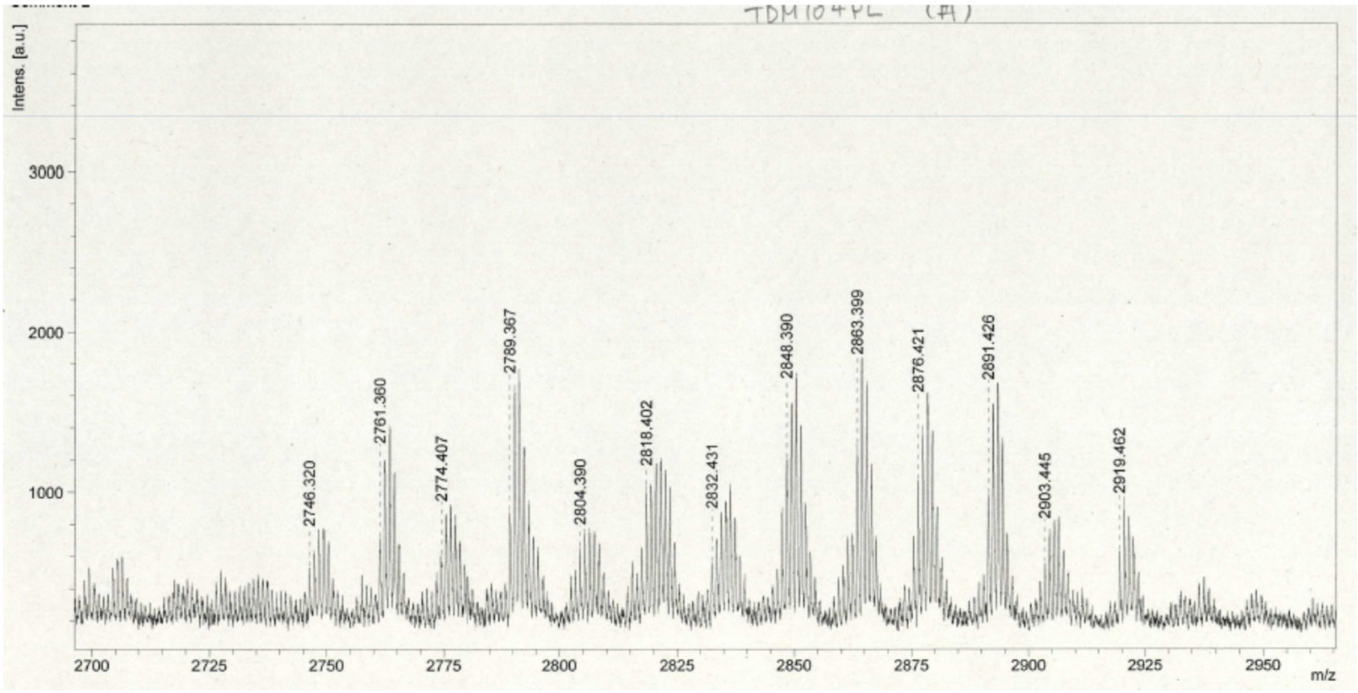

Supplementary Fig. S7. Matrix-assisted laser desorption/ionization time-of-flight (MALDI-TOF) mass spectra of TDM. (**A,B**) The samples extracted from spots #8 (**A**; MAH OCU 806) and #16 (**B**; MAH 104) on the TLC shown in **Supplementary Fig. S6** were analysed by a Ultraflex III-MALDI-TOF Mass Spectrometer (Bruker Daltonics; Leipzig, Germany) using 2,5-dihydroxybenzoic acid as a matrix in the positive ion mode. The obtained peak patterns were in agreement with the previous report by Fujita.

Reference

1. Fujita Y. Naka T, McNeil MR, Yano I. Intact molecular characterization of cord factor (trehalose 6,6'-dimycolate) from nine species of mycobacteria by MALDI-TOF mass spectrometry. *Microbiology*. 151, 3403-3416 (2005).

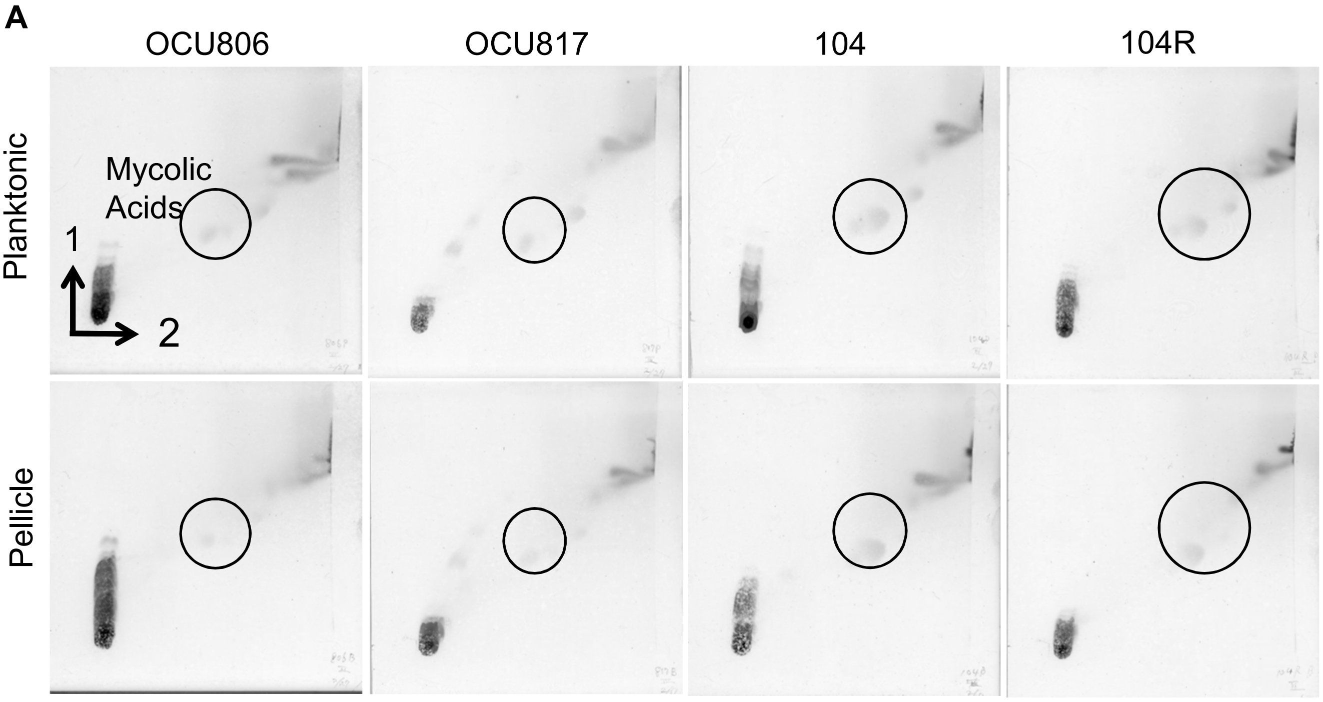

Supplementary Fig. S8. Comparison of the amount of free mycolic acids between planktonic and pellicle bacteria. The amount of total lipids derived from 1 mg of dried bacterial cells was applied to a 2D-TLC by using solvent system B (solvent 1; $CHCl_3$:MeOH, 96:4 (v/v) for 20 min, solvent 2; toluene:acetone, 80:20 (v/v) for 20 min).

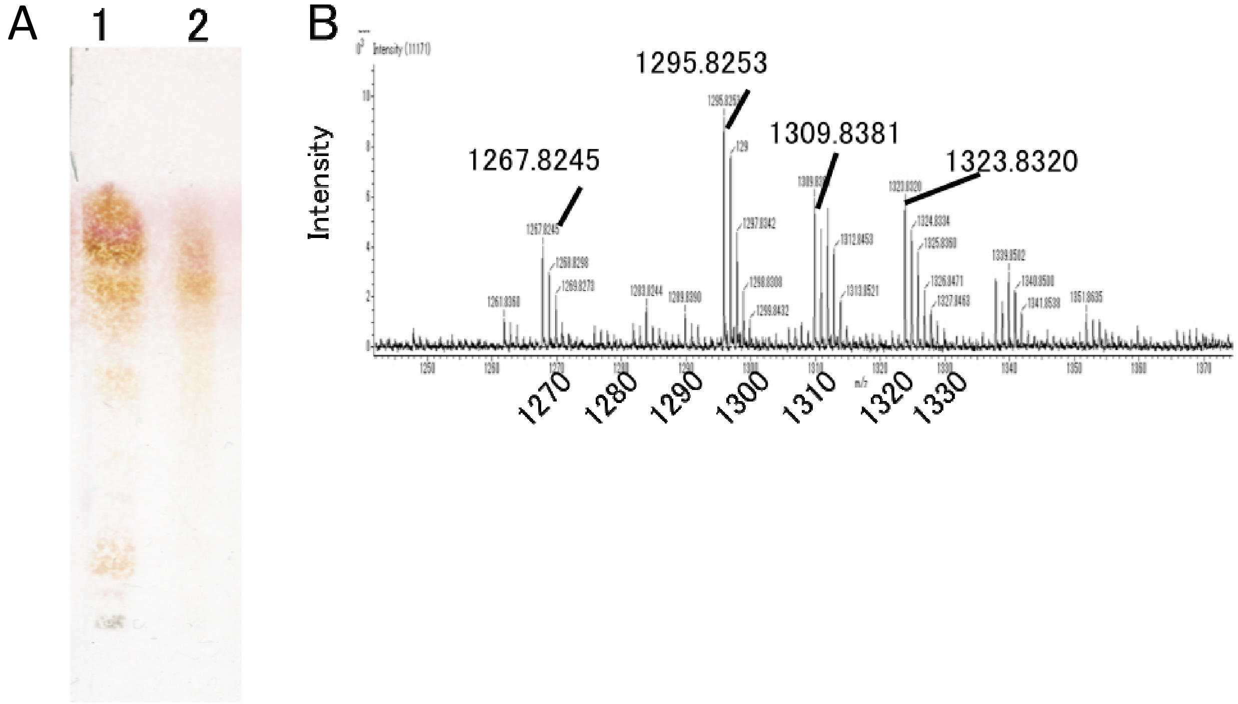

Supplementary Fig. S9. Confirmation of purified GPL. (**A**) Lipids of acetone soluble fraction (Lane 1) and purified GPL (Lane 2) extracted from MAH OCU806 on TLC. (**B**) Mass spectrum of purified GPL of MAH OCU806 obtained by ESI/MS. The separated spots on GPLs indicate difference in the number of acetyl groups at the hydroxyl sites of glycoside[1]. Apolar GPLs contain a lipopeptide core of C26-33 fatty acids amidated with D-Phe-D-alloThr-D-Ala-L-alaninol. The alloThr residue of this core is glycosylated with 6-deoxy-α-L-talose (dTal), and the alaninol residue is glycosylated with L-rhamnose (Rha). In addition, the C3 of the fatty acids is usually hydroxylated or methoxylated. Some apolar GPLs are *O*-methylated at the hydroxyl site of dTal (*O*-Me dTal)[2]. The purified GPLs are as follows; C26 fatty acyl mono *O*-acetylated GPL (m/z): $[M+Na]^+$ calcd. for $C_{68}H_{116}N_4O_{16}Na$,1267.8284; found 1267.8245, C26 fatty acyl di-*O*-acetylated GPLs (m/z): $[M+Na]^+$ calcd. for $C_{69}H_{116}N_4O_{17}Na$,1295.8233; found 1295.8253, C26 fatty acyl *O*-Me-di-*O*-acetylated GPLs (m/z): $[M+Na]^+$ calcd. for $C_{70}H_{118}N_4O_{17}Na$,1309.8390; found 1309.8381, and C27 fatty acyl di-*O*-acetylated GPL (m/z): $[M+Na]^+$ calcd. for $C_{71}H_{120}N_4O_{17}Na$,1323.8546; found 1323.8320.

References

1. Nishiuchi Y, Kitada S, Maekura R. Liquid chromatography/mass spectrometry analysis of small-scale glycopeptidolipid preparations to identify serovars of *Mycobacterium avium-intracellulare* complex.*Appl Microbiol*. **97**,738-48 (2004).
2. Pang L, Tian X, Pan W, Xie J. Structure and function of mycobacterium glycopeptidolipids from comparative genomics perspective. *J Cell Biochem*. **114**, 1705-13 (2013).
